# Inhibition of Low-Grade Inflammation by Anthocyanins after Microbial Fermentation in Vitro

**DOI:** 10.3390/nu8070411

**Published:** 2016-07-02

**Authors:** Sabine Kuntz, Clemens Kunz, Eugen Domann, Nora Würdemann, Franziska Unger, Andreas Römpp, Silvia Rudloff

**Affiliations:** 1 Department of Pediatrics, Justus Liebig University, Feulgenstrasse 12, Giessen D-35392, Germany; silvia.rudloff@ernaehrung.uni-giessen.de; 2 Institute of Nutritional Science, Justus Liebig University, Wilhelmstrasse 20, Giessen D-35392, Germany; clemens.kunz@ernaehrung.uni-giessen.de; 3 Institute of Medical Microbiology, German Centre for Infection Research, DZIF Partner Site Giessen-Marburg-Langen, Justus Liebig University, Schubertstrasse 81, Giessen D-35392, Germany; eugen.domann@mikrobio.med.uni-giessen.de (E.D.); nora.wuerdemann@hno.med.uni-giessen.de (N.W.); 4 Institute of Inorganic and Analytical Chemistry, Justus Liebig University, Schubertstrasse 60, Giessen D-35392, Germany; franziska.unger@anorg.chemie.uni-giessen.de (F.U.); andreas.roempp@uni-bayreuth.de (A.R.); 5 Chair of Bioanalytical Sciences and Food Analysis, University of Bayreuth, Frankengut D-95703, Germany

**Keywords:** grapes and berries, anthocyanins, low-grade inflammation, fermentation, in vitro co-culture model

## Abstract

The anti-inflammatory effects of anthocyanins (ACNs) on vascular functions are discussed controversially because of their low bioavailability. This study was performed to determine whether microorganism (MO)-fermented ACNs influence vascular inflammation in vitro. Therefore, MO growth media were supplemented with an ACN-rich grape/berry extract and growth responses of *Escherichia coli*, *E. faecalis* and *H. alvei*, as well as ACN fermentation were observed. MO supernatants were used for measuring the anti-inflammatory effect of MO-fermented ACNs in an epithelial-endothelial co-culture transwell system. After basolateral enrichment (240 min), endothelial cells were stimulated immediately or after 20 h with TNF-α. Afterwards, leukocyte adhesion, expression of adhesion molecules and cytokine release were measured. Results indicate that *E. coli*, *E. faecalis* and *H. alvei* utilized ACNs differentially concomitant with different anti-inflammatory effects. Whereas *E. coli* utilized ACNs completely, no anti-inflammatory effects of fermented ACNs were observed on activated endothelial cells. In contrast, ACN metabolites generated by *E. faecalis* and *H. alvei* significantly attenuated low-grade stimulated leukocyte adhesion, the expression of adhesion molecules E-selectin, VCAM-1 and ICAM-1 and cytokine secretion (IL-8 and IL-6), as well as NF-κB mRNA expression with a more pronounced effect of *E. faecalis* than *H. alvei*. Thus, MO-fermented ACNs have the potential to reduce inflammation.

## 1. Introduction

Consumption of fruit and vegetables has been associated with a reduced risk of inflammation-associated diseases, such as atherosclerosis, obesity, diabetes and metabolic syndrome [1,2]. Most of these diseases have been associated with low-grade inflammation (LGI), which is characterized by an enhanced circulation of cytokines (TNF-α, IL-6) and chemokines (IL-8), resulting in chronic activation of the vascular epithelium and recruitment of mononuclear cells [3]. The activation of endothelial cells during inflammatory processes is a well-known multistep process in which multiple cell adhesion molecules are expressed in a time-dependent manner [4]. The cell adhesion molecule E-selectin allows the first contact between mononuclear cells and the vascular endothelium during inflammation [5,6]. Thereafter, molecules of the immunoglobulin superfamily, mainly intercellular cell adhesion molecule-1 (ICAM-1) and vascular cell adhesion molecule-1 (VCAM-1), are responsible for the firm adhesion and transmigration of the cells into the tissue. The movement of mononuclear cells as a result of dysregulated cell adhesion molecule expression is a key process in the onset and progression of endothelial dysfunction. Interestingly, it has been reported that VCAM-1 plays a major role in the initiation of atherosclerosis [7]. Therefore, the development of strategies to influence this multistep process and thus prevent inflammation-associated diseases is a great challenge.

Epidemiological observation studies and intervention trials have suggested that fruit or vegetable consumption provides anti-inflammatory and anti-adhesive effects beneficial for vascular function [8,9,10,11]. Lingonberries were shown to prevent diet-induced obesity and low-grade inflammation in mouse models [12], and a healthy diet with fresh fruits and vegetables was associated with a lower endothelial dysfunction and LGI in humans [13]. Such effects of fruits, especially berries, have been mainly attributed to the presence of polyphenols with anthocyanins (ACNs) and phenolic acids (PAs) as the most abundant components [14]. The predominant ACNs identified in berries, especially in red grapes, include the 3-*O*-monoglucosides and the 3-*O*-acylated monoglucosides of cyanidin, delphinidin, petunidin, peonidin and malvidin. However, in recent years, there has been increasing evidence that not only the parental ACNs were responsible for the health effects, but also their fermented degradation products. Although ACNs can be directly absorbed in the intestine without prior chemical modification [15], plasma levels of intact ACNs were found in nmol concentrations, indicating that their bioavailability is extremely low [16,17]. Nevertheless, recently-published data suggest that intense MO fermentation in the colon generates metabolites responsible for the anti-inflammatory effects seen in vivo [18,19,20]. Gut MO populations could use ACNs or further metabolites as growth substrates. Although evidence related to the effects of ACNs on gut microbiota is still scarce and inconclusive, it has recently been shown that polyphenols are able to modulate the microbiota growth kinetics [21,22,23,24,25,26].

Consequently, colonic catabolism of ACNs may play a major role in the production of new phenolic derivatives or degradation products, which may be absorbed and exert functions initially considered to be due to the parent compounds. ACNs and their metabolites could have a high potential to reduce inflammatory markers, such as cytokines, intercellular adhesion molecule (ICAM-1), vascular cell adhesion molecule (VCAM-1) and E-selectin on the surface of TNF-α-activated endothelial cells in this context; gut microbiota may play an important role in generating possible fermentation products affecting vascular health.

Considering that ACNs and colonic catabolites of polyphenols may have different bioactivity, the aim of the present study was to investigate the microorganism-ACN interactions involved in the colonic metabolism of ACNs used in an in vitro co-culture model with epithelial cells mimicking the intestinal layer and endothelial cells mimicking the vascular side. Therefore, after batch culture fermentation of an ACN-rich grape/berry extract with different human commensals (*Escherichia coli*, *Enterococcus faecalis* and *Hafnia alvei*), changes in (1) the growth responses of these commensals; (2) ACN fermentation, as well as (3) the protective effects of fermented ACNs on low- and high-grade inflammation events in the in vitro epithelial-endothelial co-culture model were monitored.

## 2. Materials and Methods

### 2.1. Materials

TNF-α was obtained from R & D (Heidelberg, Germany) and Calcein-AM from Invitrogen (Heidelberg, Germany). Ethanol, methanol and formic acid were supplied by Merck KGaA (Darmstadt, Germany). Cell culture media (MCDB131) and supplements (EGF, ECGF) for human umbilical vein endothelial cells (HUVECs) were obtained from Promocell (Heidelberg, Germany) and those for Caco-2 cells (Dulbecco’s Eagle’s Minimum Essential Medium (DMEM)) from Invitrogen (Heidelberg, Germany). Fetal calf serum (FCS) was supplied by Biochrome (Berlin, Germany), lysogeny broth (LB) for the culture of Gram-negative microorganisms by Life Technologies GmbH (Darmstadt, Germany) and tryptic soy broth (TSB) for the culture of Gram-positives by Merck KGaA (Darmstadt, Germany). Human plasma fibronectin was obtained from Invitrogen (Heidelberg, Germany). All chemicals were of analytical grade and cell culture tested.

### 2.2. Methods

#### 2.2.1. Anthocyanin-Rich Grape/Bilberry Extract

The ACN-rich grape/berry extract made of red grapes (80/20 mixture of the grape varieties *Dakapo* and *Accent*) and bilberries (*Vaccinium myrtillus*) was produced at Geisenheim University (Section of Wine Analysis and Beverage Technology). Briefly, grapes were extracted in a press, and the resulting juice was separated, blended with bilberry juice from concentrate and pasteurized. The grape-bilberry juice was loaded onto a pilot glass column (Kronlab/Schott, 200 × 750 mm) filled with 16 L of adsorber resin (Resindion/Mitsubishi, Mailand, Italy). Water-soluble juice constituents like sugars, organic acids and minerals were washed out with two bed volumes of distilled water. The first eluate was slightly colored due to low ACN levels. The ACNs and colorless polyphenols were eluted with two bed volumes of 96% ethanol. The ethanolic fraction was concentrated by a rotary evaporator and lyophilized. ACNs were analyzed by HPLC-PDA/ESI-MS (high-performance liquid chromatography/photo diode array detection/electrospray ionization mass spectrometry); their content is given in Table 1.

#### 2.2.2. Determination of ACNs and Metabolites in In Vitro Samples Using UPLC and MS Measurement

Ultra-performance liquid chromatography (UPLC) separation was performed using an UltiMate 3000 RSLC (Rapid Separation LC) system (Dionex, Idstein, Germany) equipped with a pump (HPG 3200 RS), an autosampler (WPS-3000 TRS) and a 100 mm × 2.1 mm ID, 2.6-µm particle size, Kinetex reversed phase (C18) column (Phenomenex, Aschaffenburg, Germany). Mobile phases consisted of 0.7% v:v formic acid in water (Solvent A) and methanol (Solvent B). The solvent gradient was 2% B at 0–0.5 min, 7% B at 1 min, 15% B at 5 min, 40% B at 10 min, 70% B at 13–16 min at a flow rate of 400 µL/min (*p*(max) = 550 bar). The injection volume was 5 µL. The UPLC system was coupled to an Exactive™ Orbitrap mass spectrometer (Thermo Fisher Scientific, Bremen, Germany) by an electrospray ion source. Capillary temperature was set to 350 °C; spray voltage in positive ion mode was 4.4 kV and in negative ion mode 3.5 kV. Measurements were performed by continuously switching between positive and negative ion mode. Mass spectra were acquired in the range of *m*/*z* = 200–850 for positive ion mode and *m*/*z* = 100–850 for negative ion mode.

#### 2.2.3. Commensal Microorganisms and Culture Conditions

The influence of the ACN-rich grape/berry extract on the growth behavior of commensal microorganisms (MO) was first screened for 35 bacterial species belonging to the genera *Bacillus*, *Bacteroides*, *Branhamella*, *Citrobacter*, *Enterobacter*, *Enterococcus*, *Escherichia*, *Hafnia*, *Klebsiella*, *Lactobacillus*, *Lactococcus*, *Listeria*, *Morganella*, *Pediococcus*, *Proteus*, *Providencia*, *Serratia*, *Salmonella*, *Shigella*, *Staphylococcus* and *Yersinia*. The supplementation of the growth media with ACN-rich grape/berry extract revealed three phenotypes: (I) growth restriction; (II) growth promotion; and (III) no impact on growth. As the trait “pathogenic” or “non-pathogenic” was not associated with the observed phenotype, we performed the experiments with non-pathogenic bacteria representing each of the three phenotypes, i.e., with the Gram-positive *Enterococcus faecalis* (*E. faecalis*; Symbioflor 1; Phenotype I), as well as with the Gram-negative *Escherichia coli* (*E. coli*; K12; Phenotype II) and *Hafnia alvei* (*H. alvei*; wild-type; Phenotype III). *H. alvei* and *E. coli* were cultured with gentle shaking in a conical flask in lysogeny broth (LB; Life Technologies GmbH, Darmstadt, Germany) under aerobic conditions at 37 °C, whereas *E. faecalis* was cultured in tryptic soy broth (TSB; Merck, Darmstadt, Germany) under the same conditions. Briefly, a pure colony was used to inoculate 10 mL of broth in a 100-mL flask and incubated under vigorous shaking at 37 °C overnight. The overnight cultures were diluted 1:50 in the medium containing the ACN-rich grape/berry extract (50 µmol/L malvidin-3-glucoside (Mal-3-glc)-equivalents) or in medium without ACNs (control). The growth of these commensal MOs was assayed in time-dependent experiments by determining the optical density (OD) at 600 nm using a UV-VIS-spectrophotometer (Shimadzu UV 1001). Three different time points were examined: the lag-phase (1); the logarithmic phase (log-phase) (2); and the stationary phase (stat-phase) (3). After centrifugation of 1-mL aliquots in a Biofuge-15 centrifuge (Heraeus, Hanau, Germany) at 10,000 rpm for 10 min, the MO-free supernatant was filtered through a 0.2-µm polyethersulfone membrane (PES) Whatman filter (Sigma-Aldrich, Munich, Germany), acidified with 0.1% formic acid/H_2_O to pH 2.0 in order to reduce storage degradation. The aliquots were stored at −80 °C until further analysis (quantification of ACNs and degradation products (see “UPLC and MS Measurement for In Vitro Samples”) and the application to the in vitro system (see “Establishment of an Epithelial-Endothelial Co-Culture System”).

#### 2.2.4. Establishment of an in vitro Epithelial-Endothelial Co-Culture System

In order to investigate the anti-inflammatory effects of MO-fermented ACNs and their metabolites on endothelial cells, we developed an in vitro co-culture system with human intestinal epithelial cells (Caco-2) and human umbilical vein endothelial cells (HUVECs), as previously published [27]. Caco-2 cells were grown on semipermeable filters over 21 days in order to differentiate and develop a small intestinal cell-like phenotype. After differentiation, transwell filters were inserted onto a 24-well plate, where HUVECs had been cultured on the bottom of the cavity. After equilibration, epithelial cells in the upper compartment were exposed to the supernatant of the MO batch culture collected at lag- (1), log- (2) or stat-phase (3). Two hundred forty minutes later, inserts were removed, and HUVECs were used immediately (short-term incubation) or further incubated for 20 h (long-term incubation). To mimic LGI or high-grade inflammation (HGI), HUVECs were incubated for 3 h with 1 or 10 ng/mL TNF-α, respectively. After TNF-α stimulation, we determined: (1) leucocyte adhesion to HUVECs; (2) the expression of cell adhesion molecules; (3) the expression of NF-κB (RelA); and (4) the expression of the cytokines IL-6 and IL-8 (mRNA and protein expression).

**Figure 1:**
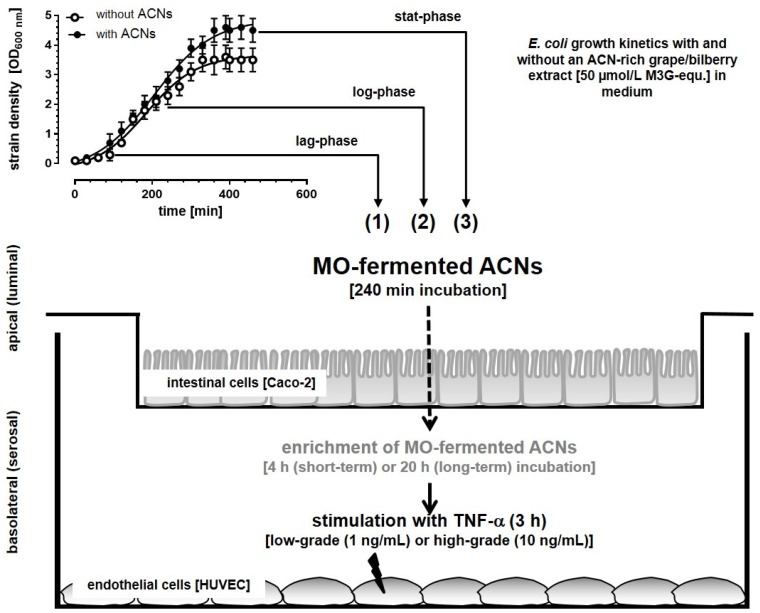
Determination of the anti-inflammatory effects of microorganism (MO)-fermented ACNs and their metabolites from lag- (1), log- (2) and stat-phase (3) in an in vitro epithelial-endothelial co-culture cell system by using, e.g., *E. coli* supernatants.

The model for investigating MO-fermented ACNs and their metabolites in the established co-culture system is shown in Figure 1.

#### 2.2.5. Epithelial and Endothelial Cells for the Co-Culture System

##### Intestinal Epithelial Cells (Caco-2 Cells)

The human colon adenocarcinoma cell line Caco-2 (HTB37™) was obtained from ATCC (ATCC/LGC standard GmbH, Wesel, Germany). The cells were routinely grown in 75 cm^2^ culture flasks using Dulbecco’s Eagle’s Minimum Essential Medium (DMEM) at pH 7.4 with 1% non-essential amino acids (NEAA) and 10% fetal calf serum (FCS). Cells were maintained in a humidified atmosphere of 5% CO_2_ in air at 37 °C. Stock passages were subcultured every four days before reaching 80%–90% confluence. For incubation studies, pre-confluent cells were trypsinized with a 0.25% (w/v) trypsin/0.53 mM EDTA solution, and 10^4^ cells were seeded onto a 24-well transwell-insert with a polycarbonate membrane (0.4-µm pore size, Becton-Dickinson, Heidelberg, Germany) and placed in a 24-well cavity. Cells were allowed to grow to confluence (2 days) and thereafter to differentiate into absorptive enterocytes in 21 days. The culture medium was changed every 2–3 days at the apical (0.5 mL) and basolateral side (1.5 mL). Alkaline phosphatase activities were measured to confirm differentiation of Caco-2 cells to an intact intestinal monolayer [27]. Furthermore, for all transport experiments, the transepithelial electrical resistance (TEER), a measure of the integrity of polarized epithelial cell monolayers, was determined before and after the experiments by using a Millicell^®^ ERS volt-ohmmeter (Millipore Corporation, Bedford, MA, USA). A TEER value ≥ 550 Ω per cm^2^ was used as an indicator for an intact epithelial layer [27].

##### Endothelial Cells

HUVECs were obtained from Promocell (Heidelberg, Germany) from pooled donors (up to four different umbilical cords). Endothelial cells were cultured on 75-cm^2^ flasks at 37 °C, 5% CO_2_ in MCDB131 (Promocell) supplemented with 10% FCS (Biochrom, Berlin, Germany), 1 µg/mL EGF and 30 µg/mL ECGF (Promocell). After reaching 80% confluence, cells were passaged at a 1:3 split ratio with trypsin/EDTA (0.05%/0.01%) solution and were used until Passages 2–4. Trypsinized cells (1 × 10^5^) were seeded on fibronectin-coated (Biochrom, Berlin, Germany) 24-well plates and allowed to grow until confluence (2 days) to be further used in co-culture experiments.

##### Incubation Studies with MO-Fermented ACNs in the Co-Culture System

For incubation studies, the medium from differentiated Caco-2 cells on transwell filters was decanted, and inserts with cells were carefully washed with the medium at 37 °C and transferred to a 24-well plate. Apical and basolateral compartments were filled for equilibration (30 min, 37 °C) with 0.5 and 1.5 mL medium, respectively. After equilibration, supernatants from batch cultures (with and without ACNs) from the lag-, log- and stat-phase were added to the apical compartment with Caco-2 cells to allow transport of metabolites to the basolateral compartment where HUVECs were attached (compare Figure 1).

In a first set of experiments, media of apical and basolateral compartments were taken after 0, 120 and 240 min, mixed with 1% formic acid/H_2_O to prevent further degradation and stored immediately at −80 °C until further analysis. Apical compartments were analyzed without solid-phase extraction (SPE) with UHPLC-MS/MS, and basolateral compartments were subjected to solid-phase extraction (SPE) cartridges on octadecylsilane (HLB; Waters Corp., Milford, MA, USA) following the method described previously [28].

In a second set of experiments, cell viability was investigated. To exclude the cytotoxic effects of the incubation medium, cell counts and cell viability were determined by using the Guava^®^ ViaCount™ assay on the Guava EasyCyte flow cytometer (Millipore Merck, Billerica, MA, USA). We measured cell viability before (0 h) and after incubation of HUVECs with cell media from the basal compartment (4 h) to ensure the viability of HUVECs during co-cultivation with intestinal cells. In all cases, viability remained between 85% and 90%. Furthermore, after each incubation experiment (short-term and long-term incubation and TNF-α-stimulation), HUVEC cell viability was tested concomitantly with the expression of cell adhesion molecules (see “Analysis of Cell Adhesion Molecules by FACS Analysis”) to ensure that there was no cytotoxic effect by possible metabolites from test substances [27].

In a third set of experiments, transwell inserts with Caco-2 cells were removed after 240 min of incubation. HUVECs were either used immediately or cultured for additional 20 h in order to evaluate a short or long time effect of ACNs and their possible metabolites. Thereafter, HUVECs were incubated with 1 ng/mL TNF-α for 3 h to mimic an LGI or with 10 ng/mL TNF-α to mimic an HGI. After endothelial activation with TNF-α, anti-inflammatory effects were measured.

#### 2.2.6. Anti-Inflammatory Effects

##### Leukocyte Cell Adhesion Assay

To evaluate leukocyte adhesion to HUVECs, we isolated leukocytes as previously described [29]. Leukocytes (0.5 × 10^6^ cells/mL) were incubated for 15 min with 1 µmol/L Calcein-AM (Invitrogen, Heidelberg, Germany). After intracellular hydrolysis to calcein, leukocytes were washed twice with pre-warmed GIBCO^®^ Hank’s Balanced Salt Solution (HBSS) to remove extracellular Calcein-AM solution. Centrifuged cells (5 min, 1500× *g*) were resuspended in 600 µL HBSS and immediately used for adhesion studies. Therefore, medium from TNF-α activated HUVECs of the co-culture system was removed and replaced by 600 µL HBSS containing calcein-dyed leukocytes (0.5 × 10^6^ cells/mL). After a 5-min incubation under shaking (150 rpm/min, 37 °C), the leukocyte suspension was aspirated, and the wells were washed twice by HBSS to eliminate leukocytes only loosely attached to endothelial cells. Firm adhesion was recorded by measuring the fluorescence of calcein-dyed cells at 538 nm after excitation at 485 nm using a fluorescence multiwell-plate reader (Fluoroskan Ascent, Labsystems, Bornheim-Hersel, Germany). A calibration curve using 1 × 10^3^–5 × 10^5^ cells was established to quantify cell adhesion. In each set of experiments samples without ACNs in the growth medium (lag-, log- and stat-phase) served as their own controls. The effect of fermented ACNs on leukocyte adhesion to TNF-α stimulated HUVECs (*n* = 3, each done in duplicate) was calculated as percent of the adhesion to simulated HUVECs without ACNs in the media (positive control, which was set to 100%).

##### Analysis of mRNA Expression of Cell Adhesion Molecules and Cytokines by Real-Time PCR

After the incubation experiments described above, mRNA from HUVECs was isolated using the Dynabeads mRNA DIRECT™ kit (Life Technologies, Darmstadt, Germany) according to the manufacturer’s instructions. cDNA synthesis was carried out in a reaction volume of 20 µL containing 500 ng RNA, 50 mM Tris-HCl (pH 8.3), 75 mM KCL, 3 mM MgCl_2_, 10 mM dithiothreitol, 100 ng oligodeoxythymidine 15 primer, 500 µM of each deoxynucleotide triphosphate (dATP, dGTP, dCTP and dTTP), 10 U ribonuclease inhibitor and 200 U MulV (Moloney murine leukemia virus reverse transcriptase); all reagents were obtained from Invitrogen. The samples were incubated at 37 °C for 60 min, followed by an incubation at 95 °C for 15 min. mRNA expression of target genes was measured using the 7500 Real-Time PCR System (Applied Biosystems, Darmstadt, Germany). The gene-specific primers and probes used in this study (Table 2) were designed using the sequences accessible in the NCBI Reference Sequence and the Primer Express software 3.0 (Applied Biosystems, Foster City, CA, USA). All probes were labeled with the fluorescent dyes 5-FAM (6-carboxy-fluorescein) as the reporter and 3-TAMRA (6-carboxy-tetramethyl-rhodamine) as the quencher. Primers and probes were purchased from Sigma (Deisenhofen, Germany). A total reaction volume of 25 µL contained 1× reaction buffer with 250 nmol of each primer, 150 nmol probe and 1 µL cDNA. The PCR running conditions were: 10 min of initial denaturation at 95 °C, followed by 45 cycles of 30 s at 95 °C, 30 s at 58 °C for annealing, 30 s at 60 °C and 15 s at 75 °C. Samples were run in triplicate. Real-time RT-PCR results are shown as the relative expression level of normalized samples (Δ cycle threshold (C_t_)) in relation to the expression of the calibrator sample (2^−ΔΔCt^). The C_t_ value refers to the cycle number at which the amplification curve crosses the threshold line; ΔC_t_ is calculated by subtracting the C_t_ value of the corresponding housekeeper gene GAPDH (endogenous reference control) from the specific C_t_ value of the target gene, and ΔΔC_t_ is obtained by subtracting ΔC_t_ of each experimental sample by the ΔC_t_ of the calibrator. In each set of experiments, samples without ACNs in the growth medium (lag-, log- and stat-phase) served as their own controls. Relative expression levels (*n* = 3, each done in duplicate) in TNF-α-stimulated HUVECs without ACNs in the media were used as the positive control; the effects of ACNs on the expression levels of cell adhesion molecules and cytokines were then calculated in relation to the positive control, which was set to 100%. The inhibition of expression (samples with ACNs in growth medium) was compared in relation to the positive control.

##### Analysis of Cell Adhesion Molecules by Flow Cytometry

After stimulation of HUVECs with TNF-α for 3 h, cells were washed twice with cold PBS, removed by careful trypsinization and pelleted (150× *g*, 5 min). Following two washes with FACS buffer (Ca/Mg-free PBS with 0.5% FCS), cells were incubated with FS (fluorescein)-conjugated mouse monoclonal anti-human ICAM-1 (CD54) and anti-human E-selectin (CD62E), as well as with PE (phycoerythrin)-conjugated mouse monoclonal anti-human VCAM-1 (CD106) (R&D Systems, Heidelberg, Germany) for 1 h at 4 °C. Isotype controls for PE (mouse IgG_2a_ isotype control PE) and FS (mouse IgG_1_ isotype control FS) were used as negative controls. For each test, 10 µL PE- or FS-conjugated antibodies per well were incubated and analyzed by flow cytometry in a cytofluorometer (Millipore GmbH, Schwalbach, Germany). Quantification of adhesion molecule expression was obtained using CytoSoft Version 4.2.1 software (Millipore, Darmstadt, Germany) by comparing mean fluorescence intensities (MFI). In each set of experiments samples without ACNs in the growth medium (lag-, log- and stat-phase) served as their own controls. MFI of TNF-α stimulated HUVECs without fermented ACNs in the media was set to 100% and used as the positive control; the effect of ACNs on the expression of cell adhesion molecules was calculated in relation to the positive control (*n* = 3, each done in duplicate).

##### Cytokine Determination

IL-6 and IL-8 concentrations present in the supernatants of TNF-α-stimulated HUVECs were measured using RayBio immunoassays (BioCat GmbH, Heidelberg, Germany) and the Digiscan Reader (Asys Hitech GmbH, Eugendorf, Austria) according to the manufacturer’s instructions. IL-6 and IL-8 protein expression was calculated using Microwin software Version 2.15 (Asys Hitech, Eugendorf, Austria). In each set of experiments, samples without ACNs in the growth medium (lag-, log- and stat-phase) served as their own controls. Il-6 and IL-8 protein concentrations of TNF-α-stimulated cells were set to 100% and used as the positive control, and the inhibition of protein expression (samples with ACNs in growth medium) was compared to the positive control (*n* = 3, each done in duplicate).

#### 2.2.7. Statistical Analysis

We have investigated each effect in three independent experiments, and each experiment was conducted at least with duplicates. Results are presented as the mean values ± SEM, the mean values ± SD or relative to controls (%) as indicated (*n* = 3). Data were statistically evaluated using GraphPad Prism software Version 6.0.7 (La Jolla, CA, USA); differences between and within experimental groups were analyzed using the two-tailed unpaired Student’s *t*-test and ANOVA, respectively. Differences were accepted as statistically significant at a *p*-value < 0.001 ***, < 0.01 ** or < 0.05 *.

## 3. Results

### 3.1. Microbial Growth Response and ACN Fermentation

**Figure 2:**
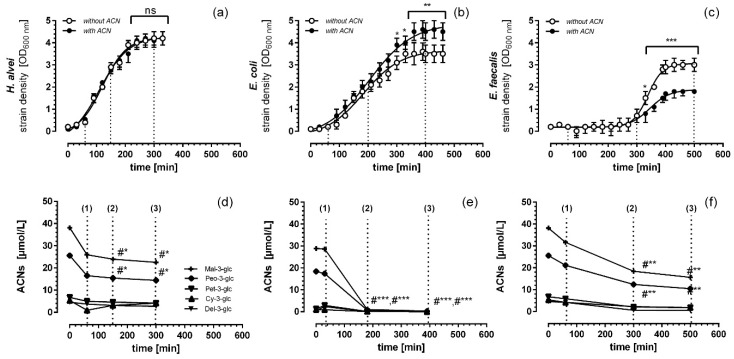
MO growth kinetics of *H. alvei* (**a**) *E. coli* (**b**) and *E. faecalis* (**c**) with or without ACNs in culture media. MO growth media were supplemented with 50 µmol/L malvidin-3-glucoside (Mal-3-glc) equivalents of an ACN-rich grape/berry extract or remained non-supplemented. The growth of the MOs was monitored spectrophotometrically (OD_600_ nm) and was plotted over time (min). At different time points (lag-phase (1), log-phase (2) and stat-phase (3)), MO suspensions with and without ACNs were taken and ACNs in the culture media of *H. alvei*, *E. coli* and *E. faecalis* were determined as described in the Materials and Methods (**d**–**f**). Values are given as the means ± SEM (*n* = 3, each done in duplicate). Differences between growth kinetics with and without ACNs, as well as between ACN fermentation and the initial ACN concentration in the growth media were significant at * *p* < 0.05, ** *p* < 0.01 and *** *p* < 0.005 and #* *p* < 0.05, #** *p* < 0.01 and #*** *p* < 0.005, respectively.

The ACN-rich extract of grapes and bilberries was tested for growth properties and MO activities against Gram-positive *E. faecalis* and Gram-negative *E. coli* and *H. alvei*. The selected microorganisms are commensals inhabiting the gastrointestinal tract of humans [30]. As shown in Figure 2, the three MO commensals exhibited different growth responses. Growth of *H. alvei* was not affected by ACNs in the media (Figure 2a), whereas that of *E. coli* was significantly enhanced in the presence of ACNs in the growth medium (Figure 2b). In *E. faecalis*, however, ACNs caused a significant growth inhibition (Figure 2c). For both Gram-negatives, *H. alvei* and *E. coli*, the lag-phases were very short, indicating a quick adaptation to the different growth conditions, whereas *E. faecalis* required significantly more time to adapt (Figure 2).

In order to evaluate whether ACNs were utilized by the MOs, concentrations of the main ACNs were determined in the lag-, log- and stat-phase of growth responses (Figure 2d–f). In contrast to *H. alvei* and *E. faecalis*, ACNs and their major components, malvidin-3-glucoside (Mal-3-glc) and peonidin-3-glucoside (Peo-3-glc), were rapidly fermented by *E. coli*, i.e., after 180 min of cultivation, no ACNs were detected in the growth media. In the case of *H. alvei* and *E. faecalis*, the decrease of ACNs in the growth media became significant after 150 min and 300 min, respectively. Although we measured a considerable decrease in ACN concentration, no specific degradation product could be determined.

### 3.2. Apical ACN-Degradation after Incubation of Co-Cultured Caco-2 and HUVECs with Batch Cultures from the Lag-, Log- and Stat-Phase

To evaluate whether MO-fermented ACNs were transported across the Caco-2 monolayers, apical degradation and basolateral enrichment of ACNs or their metabolites were measured with UHPLC-MS/MS during the 240-min incubation time of the co-culture in vitro system (Table 3).

ACN supernatants from the lag-, log- and stat-phase after MO fermentation were tested for degradation with Caco-2 cells cultured on transwell inserts in the in vitro system (pH 7.4). As shown in Table 3, after 240 min of incubation of ACNs with *H. alvei*, the losses of the major ACNs, Mal-3-glc and Peo-3-glc, compared to their initial concentrations were significant after incubation with metabolites generated during the lag- and log-phase of growth. Similar results after 240 min were observed for *E. faecalis* (Table 3). In contrast to *E. faecalis* and *H. alvei*, only in the supernatants derived from the lag-phase of *E. coli*, intact ACNs were observed, and the loss of Mal-3-glc was 50% ± 7% and that of Peo-3-glc was 55% ± 7% after incubation for 240 min. Neither in the log-phase nor in the stat-phase were ACNs detected in the supernatants (Table 3). Furthermore, neither ACNs nor their metabolites could be determined in the basolateral compartment of the transwell systems (compare Figure 1).

### 3.3. Inhibition of Leukocyte Adhesion to HUVEC by MO-Fermented ACNs

To investigate whether ACNs fermented by *E. faecalis*, *E. coli* and *H. alvei* have any anti-inflammatory effect on the human vascular system, we examined the effects of these metabolites from the lag-, log- and/or stat-phase on leukocyte adhesion to TNF-α-activated HUVECs in comparison to control media without ACNs. Therefore, supernatants collected during the lag-, log- and stat-phase from MO batch culture fermentations (with and without ACNs) were subjected to Caco-2 cells cultured on transwell inserts.

After 240 min of incubation, the insert with Caco-2 cells was removed, and basolateral solutions were used immediately or incubated for another 20 h with HUVECs cultured on the bottom of the basolateral compartment (compare Figure 1). Thereafter, HUVECs were stimulated for 3 h with 1 or 10 ng/mL TNF-α. 

**Figure 3:**
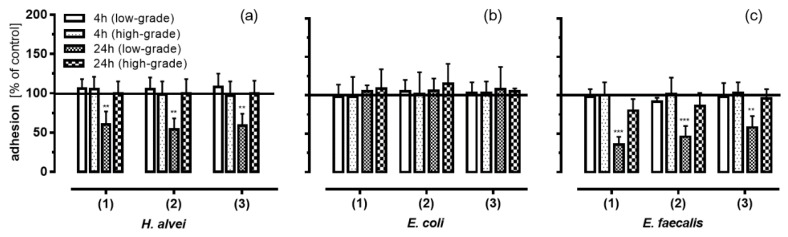
Anti-adhesive effect of ACNs after MO-fermentation on leucocyte adhesion to HUVECs under inflammatory conditions by (**a**) *H. alvei*; (**b**) *E. coli* and (**c**) *E. faecalis*. After incubation of Caco-2 cells with fermented ACNs from the lag- (1), log- (2) and stat-phase (3), transwell inserts were removed, and the co-cultured HUVECs were used immediately (4 h) or were further incubated in the basolateral compartment (20 h). Thereafter, cells were stimulated for 3 h using 1 ng/mL TNF-α (low-grade inflammation) or 10 ng/mL TNF-α (high-grade inflammation). After TNF-α stimulation, leukocyte adhesion was measured as described in the Materials and Methods section. Corresponding controls (maximal adhesion to HUVECs after low-grade or high-grade TNF-α stimulation) were set to 100%. Values are given as the mean ± SD (*n* = 3). Differences were significant at ** *p* < 0.01 and *** *p* < 0.001).

Figure 3 shows that leukocyte adhesion to HUVECs was significantly reduced by ACNs after fermentation by *H. alvei* and *E. faecalis* when HUVECs were pre-incubated for 24 h prior to low-grade TNF-α stimulation (1 ng/mL; Figure 3a,c); neither short-term pre-incubation (4 h) nor high-grade inflammatory conditions (10 ng TNF-α mL) had any effect. *H. alvei* and more pronounced *E. faecalis* obviously generated metabolites that were able to inhibit leukocyte adhesion to TNF-α-stimulated HUVECs. In the case of ACNs fermented by *H. alvei*, these effects were independent of the possible metabolites generated in the (1) lag-, (2) log- or (3) stat-phase. Pre-incubation of metabolites generated by *E. faecalis* revealed a more pronounced effect, with a greater effect induced by metabolites generated during the lag- and log-phase compared to those from the stat-phase. Interestingly, supernatants from *E. coli*-fermented ACNs did not influence leukocyte adhesion, neither after long- and short-term incubation nor under low- or high-grade inflammatory conditions (Figure 3b).

### 3.4. Inhibition of Adhesion Molecules Expression on HUVECs under Low-Grade Inflammatory Conditions by Fermented ACNs

**Figure 4:**
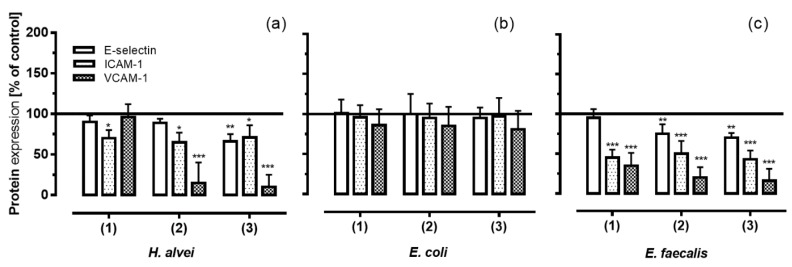
Expression of the adhesion molecules E-selectin, ICAM-1 and VCAM-1 on HUVECs under low-grade inflammation (LGI) and long-term incubation conditions fermented by (**a**) *H. alvei*; (**b**) *E. coli* and (**c**) *E. faecalis*. After incubation of Caco-2 cells with fermented ACNs from the lag- (1), log- (2) and stat-phase (3), transwell inserts were removed, and the co-cultured HUVECs were used after 20 h. Thereafter, cells were stimulated for 3 h using 1 ng/mL TNF-α (LGI). After TNF-α stimulation, protein expression (MFI, main fluorescence intensity) was measured as described in the Materials and Methods section. Corresponding controls (maximal expression of cell adhesion molecules after LGI) were set as 100%. Values are given as the mean ± SD (*n* = 3). Differences were significant at * *p* < 0.05, ** *p* < 0.01 and *** *p* < 0.001.

Since our results from the adhesion assays (see Section 3.3) were significant for inhibiting low-grade inflammation and under long-term conditions, these conditions were chosen for subsequent experiments. In order to investigate which adhesion molecules were responsible for the adhesion of leukocytes to endothelial cells, we quantified mRNA and protein levels of E-selectin, ICAM-1 and VCAM-1. Figure 4 shows that long-term incubation of HUVECs with the basolateral solutions with ACNs that had been fermented by *H. alvei* and *E. faecalis* and transported through a Caco-2 cell layer resulted in a decrease of all three adhesion molecules compared to positive controls (Figure 4a,c).

ACNs fermented by *H. alvei* significantly inhibited the TNF-α-induced protein expression with a greater effect on ICAM-1 and VCAM-1 than on E-selectin. A significant inhibition of E-selectin protein levels was only observed with stat-phase-generated ACNs, whereas ACNs generated in the lag- and log-phase had no effect compared to positive controls. ICAM-1 protein expression was significantly reduced by all metabolites no matter whether they were derived from the lag-, log- or stat-phase of growth; the greatest inhibitory effect, however, was found for VCAM-1 expression.

Similar to the effects of ACNs fermented by *H. alvei*, metabolites from *E. faecalis* influenced the expression of all three adhesion molecules, although with a greater effect on ICAM-1 and VCAM-1 than on E-selectin expression (Figure 4c). Again, the greatest effect was observed for the inhibition of VCAM-1 expression; ACN metabolites generated by *E. coli* failed to induce any effect (Figure 4b).

**Figure 5:**
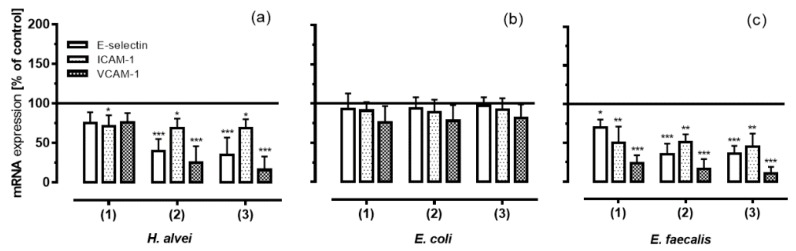
mRNA expression levels of E-selectin, ICAM-1 and VCAM-1 on HUVECs under LGI conditions and long-term incubation ACNs fermented by (**a**) *H. alvei*; (**b**) *E. coli* and (**c**) *E. faecalis*. After incubation of Caco-2 cells with fermented ACNs from the lag- (1), log- (2) and stat-phase (3), transwell inserts were removed, and the co-cultured HUVECs were further incubated for 20 h. Thereafter, cells were stimulated with 1 ng/mL TNF-α for 3 h (LGI). After TNF-α stimulation, mRNA expression levels were measured as described in the Materials and Methods section. Corresponding controls (maximal expression after LGI) were set as 100%. Values are given as the mean ± SD (*n* = 3). Differences were significant at * *p* < 0.05, ** *p* < 0.01 and *** *p* < 0.001.

Concomitant with the reduced protein levels of the adhesion molecules induced by ACN metabolites generated by *H. alvei* and *E. faecalis*, mRNA expression was influenced in a similar way, indicating that reduced protein expression was a result of reduced mRNA expression levels. Again, no effects were observed by *E. coli*-generated ACN metabolites (Figure 5).

### 3.5. Inhibitory Effects of Fermented ACNs on Cytokine Expression and Secretion in HUVECs under Low-Grade Inflammatory Conditions

**Figure 6:**
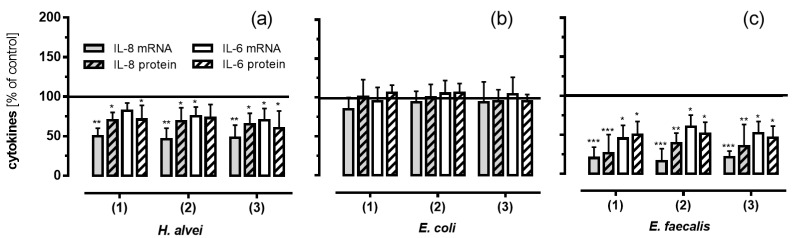
The effect of ACNs fermented by *H. alvei* (**a**); *E. coli* (**b**) and *E. faecalis* (**c**) on cytokine mRNA and protein secretion in HUVECs under LGI conditions and long-term incubation. After incubation of Caco-2 cells with fermented ACNs from the lag- (1), log- (2) and stat-phase (3), transwell inserts were removed, and the co-cultured HUVECs were further incubated for 20 h. After TNF-α stimulation, mRNA and protein expression were measured as described in the Materials and Methods section. Corresponding controls (maximal expression after LGI) were set as 100%. Values are given as the mean ± SD (*n* = 3). Differences were significant at * *p* < 0.05, ** *p* < 0.01 and *** *p* < 0.001.

To further validate the anti-inflammatory results described above, we determined the concentrations of selected cytokines, such as IL-6 and IL-8, participating in the inflammatory process. As shown in Figure 6, pre-treatment of TNF-α stimulated HUVECs with ACN metabolites generated by *H. alvei* and *E. faecalis* significantly reduced mRNA and protein levels of IL-8 and, to a lesser extent, of IL-6, whereas ACN-metabolites generated by *E. coli* did not have any effect.

Fermented ACNs from the lag-, log- and stat-phase of *H. alvei* growth significantly reduced IL-6 and IL-8 mRNA, as well as protein expression compared to controls (Figure 6a). ACNs generated by *E. faecalis* had an even stronger inhibitory effect (Figure 6c), whereas no effects were observed for *E. coli* supernatants (Figure 6b).

### 3.6. Inhibitory Effects of Fermented ACNs on NF-κB mRNA Expression in HUVECs under Different Low-Grade Inflammatory Conditions and Long-Term Incubation

**Figure 7:**
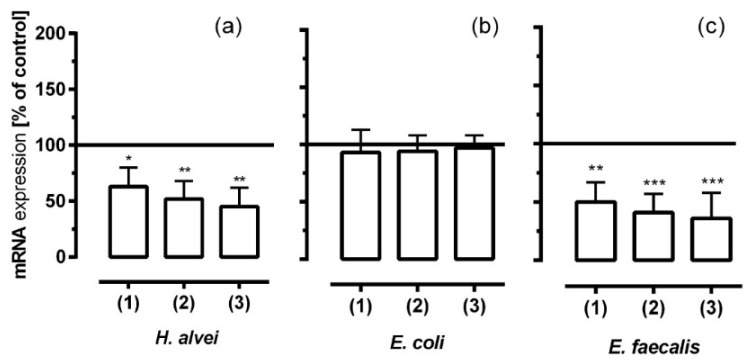
The effect of ACNs fermented by *H. alvei* (**a**); *E. coli* (**b**) and *E. faecalis* (**c**) on NF-κB mRNA expression in HUVECs under LGI conditions and long-term incubation. After incubation of Caco-2 cells with fermented ACNs from the lag- (1), log- (2) and stat-phase (3), transwell inserts were removed, and the co-cultured HUVECs were further incubated for 20 h. After TNF-α stimulation, mRNA and protein expression was measured as described in the Materials and Methods section. Corresponding controls (maximal expression after LGI) were set as 100%. Values are given as the mean ± SD (*n* = 3). Differences were significant at * *p* < 0.05, ** *p* < 0.01 and *** *p* < 0.001.

We measured the NF-κB (RelA, Reticuloendotheliosis homolog A) mRNA expression since NF-κB not only regulates the expression of cytokines, but also that of adhesion molecules under inflammatory conditions. As shown in Figure 7, pre-treatment of TNF-α-stimulated HUVECs with ACN metabolites generated from *H. alvei* and *E. faecalis* significantly reduced mRNA levels of NF-κB, whereas *E. coli*-generated ACN-metabolites did not show any effect.

Fermented ACNs from the lag- log- and stat-phase of *H. alvei* cultures significantly reduced NF-κB mRNA expression (Figure 7a). Again, ACNs from *E. faecalis* fermentation reduced mRNA levels to an even stronger extent (Figure 7c), whereas ACN metabolites from *E. coli* had no effects (Figure 7b).

## 4. Discussion

The link between the anti-inflammatory effects of dietary polyphenols in general and active molecules within this diverse group of structures has not yet been clearly established. This is partly due to open questions with regard to (1) the identification and quantification of metabolites found in vivo, i.e., in plasma after an ACN-rich diet; and (2) the molecular mechanisms they trigger within the target cells. The structural identity of ACNs as bioactive molecules in vivo has only scarcely been explored due to their intense MO fermentation and intestinal metabolism in the gut. An increasing number of reports have been published looking at methods using chemical or enzymatic synthesis to generate phase II metabolites (such as glucuronides and methylated or sulfated conjugates) or single known metabolites from degradation and fermentation pathways for testing their activity in cell culture studies [31]. Recently, we reported that ACNs from a grape/bilberry extract, as well as malvidin-3-*O*-glucoside (Mal-3-glc) were able to reduce parameters associated with LGI in an epithelial-endothelial in vitro co-culture system [27]. In the present study, we investigated the anti-inflammatory effects of ACNs in this in vitro system using MO-fermentation products of different commensal microorganisms. We were able to show for the first time that ACN metabolites generated from *E. faecalis* and *H. alvei* significantly inhibited leukocyte adhesion under LGI and long-term incubation. These effects were associated with a significant downregulation of the levels of adhesion molecules confirmed by real-time PCR and FACS analysis, as well as the inhibition of chemokine secretion and expression in endothelial cells. These inhibitory effects partially compensated the stimulation as LGI in endothelial cells completely (Figure 4, Figure 5 and Figure 6). In contrast, ACNs fermented by *E. coli* failed to inhibit cell adhesion and inflammation.

For the microorganisms themselves, ACNs seem to serve as substrates, however, with different effects on the growth kinetics of the three selected commensals. The growth of *E. coli* for example was enhanced in the presence of ACNs with concomitant reduction of ACNs, mainly Mal-3-glc and peonidin-3-*O*-glucoside (Peo-3-glc), in the growth media. In contrast, the growth of *E. faecalis* was inhibited by ACNs, and the growth of *H. alvei* was not affected at all. Although we observed decreasing concentrations of ACNs, of the major ACNs Mal-3-glc and Peo-3-glc, as well as of the minor ACNs cyanidin-, delphinidin- and petunidin-3-*O*-glucoside, we could not detect any ACN metabolites in the MO growth media during the cultivation time. It has been previously described that β-glucosidase of intestinal microbiota hydrolyzes ACNs by cleavage of the 3-*O*-linked glucose. These generated aglycones were further degraded or transported across the epithelium [32]. Similar results were reported from Sanchez-Patan et al. [22] and Hidalgo et al. [33], who identified several metabolites after the incubation of an ACN-rich extract with fecal slurry from human volunteers. Similar to our study, they observed a rapid decrease of Mal-3-glc and Peo-3-glc. Although they were not able to detect any aglycones, concomitant with the decreased ACNs, they found metabolites, such as catechol/pyrocatechol after short-term (10–30 h) and benzoic acid derivates after long-term incubation (5–30 h). Syringic acid (4-hydroxy-3,4-dimethoxybenzoic acid) as a result of Mal-3-glc B-ring degradation was observed only by Sanchez-Patan et al. and Hidalgo et al. after 5 h cultivation when ACNs were degraded. Protocatechuic (4-hydroxy-3,4-dimethoxybenzoic acid) and vanillic acid (4-hydroxy-3-methoxybenzoic acid) deriving from the B-ring degradation of cyanidin- and Peo-3-glc were not detected. Instead, they observed a significant increase of benzoic acid as a final degradation product [22,33].

Although most studies investigated the anti-MO effects of ACN activity [34,35,36], recent research focused on ACN-related changes within the different groups of intestinal microorganisms. Thus, Sanchez-Patan et al. analyzed various MO species after an ACN-rich diet and showed that *Bifidobacterium* spp. and *Lactobacillus* spp. remained constant and that *Clostridium histolyticum* was inhibited. In another study with grape seed extracts, they received similar results with an enhanced growth of *Lactobacillus*/*Enterococcus* spp. and a decrease of *Clostridium histolyticum* [24], which was also observed by Hidalgo et al. [33] using Mal-3-glc or mixed ACNs. Differences between our study and others could be attributed to the time point that the ACN-metabolites were determined, the variability in the ACN-application (concentration and presence of major ACNs), as well as to the fermentation model with a different set of microorganisms. In our fermentation model, we used single commensals in order to evaluate the MO-specific contribution to ACN fermentation and the formation of possible MO-specific metabolites. 

However, in terms of the biological activity of ACNs, the MO metabolism may at least in part be responsible for the health-promoting properties due to their anti-adhesive, anti-inflammatory or anti-oxidative potential, e.g., the inhibition of the pro-inflammatory cytokine TNF-α and the reduction of leukocyte adhesion to endothelial cells in particular are key mechanisms in the control of atherogenesis. Keeping in mind that LGI is the first step in the pathogenesis of atherosclerosis, inhibition of this step would be a great success. In this context, our results indicate that the exposure of ACN metabolites generated by *H. alvei* and *E. faecalis* to the co-cultured intestinal-endothelial cell culture model is associated with a significant reduction of the TNF-α-induced adhesion of leukocytes to HUVECs under LGI conditions. We showed that TNF-α-induced leukocyte adhesion to HUVECs was reduced following a 24-h pre-exposure to MO-derived ACN metabolites suggesting an anti-adhesive effect of MO-specific metabolites. Furthermore, this effect could only be observed, when HUVEC cells were stimulated with low doses of TNF-α mimicking an LGI and long-term pre-incubation (24 h), but not after short-term incubation (4 h). Although we observed a further decrease of ACNs in the apical compartment of the co-culture system during the incubation of intestinal cells with fermented MO culture supernatant, no ACNs, ACN-phase-II-metabolites or other metabolites were detected at the basolateral side, which was in accordance with previous studies [37,38,39]. However, it may still be possible that unknown intermediate metabolites were generated, the effects being prominent within the 24-h pre-incubation period only. Nevertheless, anti-adhesive effects exerted by metabolites generated by *E. faecalis* and *H. alvei* were found to be associated with changes in the expression levels of adhesion molecules involved in these processes, such as E-selectin, ICAM-1 and VCAM-1. Interestingly, the effect of metabolites from the stat-phase induced a greater decrease of all three cell adhesion molecules than that of metabolites from the lag-phase, which indicates that different metabolites may have been generated. The effect was particularly strong in the case of VCAM-1 and ICAM-1 and to a lesser extent on E-selectin. This was demonstrated with metabolites generated by *E. faecalis* and to a lesser extent with metabolites generated by *H. alvei*. Again, metabolites generated by *E. coli* did not show any effect. The reasons why ACN metabolites generated by *E. coli* failed to affect LGI parameters are not known. However, as shown in our study, ACNs seem to have been used by *E. coli* possibly as a growth substrate and displayed distinct growth effects (Figure 2, Table 3). The enhanced growth response indicates that ACNs may be suitable substrates for this strain and that the corresponding intermediates and/or end products were different from those of *E. faecalis* or *H. alvei* along with various vascular effects. It has recently been shown that different colonic microrganisms used Mal-3-glc or cyanidin-3-glucoside as growth substrates in different ways and generated different metabolites [25]. The authors speculate that enzymes, such as β-glucosidases, are needed to catalyze such reactions and, thus, could promote bacterial species. These observations are supported by Esplay et al. and Faria et al., showing similar results with *Bifidobacterium* spp. and *Lactobacillus* spp. [40,41]. However, the functions, mechanisms and biotransformation of ACNs to intermediates and end products remain to be investigated in vitro or in vivo in order to get more insights into the contribution of the MO community to vascular health effects.

In former studies, Hidalgo et al. have shown for possible breakdown products of ACNs, such as gallic acid, that the TNF-α-induced ICAM-1 and VCAM-1 expression levels were reduced by high concentrations of this phenolic acid (10–100 µM). It should be noted that they used TNF-α concentrations of 10 ng/mL to mimic a chronic inflammation, a 10-times higher concentration compared to what we called an LGI [42]. Under these conditions, high concentrations of gallic acid were necessary to overcome the TNF-α-induced pro-inflammatory effect in endothelial (EA.hy926, hybridoma cell line of HUVECs and A549 (lung tumor cells)) cells. Keeping in mind that the gallic acid concentration in plasma is considered to be low, the in vitro concentrations used were manifold higher than that reported under physiological conditions [43]. Chau et al. showed that 100 µg/mL purple sweet potato leaf extract and its major ACN cyanidin attenuated TNF-α-induced (2 ng/mL) VCAM-1 expression in human aortic endothelial cells (HAECs) [44]. Again, such high concentrations used in the in vitro model do not reflect the physiological situation. However, earlier studies have shown that the anti-adhesive action of flavonoids is mediated by their inhibitory effect on nuclear-factor κB (NF-κB), which is in agreement with our data. In our study, the expression of NF-κB mRNA in TNF-α-stimulated HUVECs decreased with metabolites generated by *E. faecalis* or *H. alvei*, whereas metabolites generated by *E. coli* failed to downregulate NF-κB gene expression. However, inhibition of the NF-κB signaling pathway to reduce the expression of pro-inflammatory mediators is a well-known process, and ACNs, specifically phenolic acids, have previously been shown to influence these signaling pathways in multiple ways [45], either by ROS-dependent p38 MAPK activation, NF-κB translocation and NF-κB-modulated gene expression [46] or ROS independently [47]. Furthermore, Nizamutdinova et al. have shown that ACNs not only regulate NF-κB pathways, but also differently regulate the TNF-α-mediated expression of VCAM-1 and ICAM-1 through the modulation of the GATA and IRF-1 (IFN regulatory factor-1) binding activity via Jak/STAT-3 activation [48], but data concerning possible metabolites have not been reported so far. The reduced NF-κB mRNA expression by metabolites generated by *E. faecalis* and *H. alvei* correlated with the reduced expression of the cell adhesion molecules and cytokine expression.

In our study, LGI-induced leukocyte adhesion to HUVECs was concomitant with the upregulation of IL-6 and IL-8, chemoattractant cytokines acting as potent regulators of inflammatory atherogenesis. Metabolites derived from the fermentation of ACNs by *E. faecalis* and *H. alvei*, however, effectively reduced leukocyte adhesion, as well as IL-6 and IL-8 expression; metabolites from *E. coli* were not effective.

## 5. Conclusions

Taken together, our results showed for the first time that metabolites generated by different commensals, such as *E. faecalis* and *H. alvei*, ameliorated the inflammatory response in human endothelial cells by reducing leukocyte adhesion to stimulated endothelial cells and by regulating the levels of adhesion molecules and chemotactic markers, especially of IL-8 and IL-6. Under the conditions tested (LGI and long-term incubation), these effects were significant, whereas short-term incubation under HGI was not affected. Although it is difficult to extrapolate these data to the in vivo situation, overall, our observations support the hypothesis that low-grade inflammation, which is considered to be the initial step in atherogenesis, could be inhibited by ACN fermentation products. Nevertheless, further studies are needed to identify metabolites responsible for these effects.

## Figures and Tables

**Table 1:** Anthocyanins (ACNs) in grape/berry extract. The extract was analyzed by HPLC-PDA/ESI-MS; ACN concentrations are given as mg/L and µmol/L (mean ± SD, *n* = 3).

Anthocyanins	(mg/L)	(µmol/L)
malvidin-3-glucoside	419.0 ± 1.5	849.1 ± 3.0
malvidin-3-(6″-p-coumaryl)-glucoside	102.9 ± 0.6	160.9 ± 0.9
malvidin-3-(6″-*O*-acetyl)-glucoside	87.8 ± 0.5	164.0 ± 0.9
malvidin-3-arabinoside	5.4 ± 0.2	11.7 ± 0.4
malvidin-3,5-diglucoside	5.3 ± 0.1	8.2 ± 0.2
Σ malvidin	615.1 ± 2.8	1185.7 ± 5.2
peonidin-3-glucoside	248.9 ± 0.6	537.1 ± 1.3
peonidin-3-(6″-*O*-acetyl)-glucoside	31.2 ± 0	61.7 ± 0.0
peonidin-3,5-diglucoside	7.4 ± 0.8	11.8 ± 1.3
Σ peonidin	287.5 ± 1.4	610.6 ± 2.6
delphinidin-3-glucoside	96.4 ± 1.3	207.1 ± 2.8
delphinidin-3-arabinoside	14.2 ± 1	32.6 ± 2.3
delphinidin-3-galactoside	15.5 ± 0.2	33.3 ± 0.4
delphinidin-3-(6″-*O*-acetyl)-glucoside	9.1 ± 0.3	17.9 ± 0.6
Σ delphinidin	135.2 ± 2	290.9 ± 6.1
petunidin-3-glucoside	103.4 ± 0,8	215.7 ± 1.7
petunidin-3-(6″-p-coumaryl)-glucoside	29.9 ± 0.2	47.8 ± 0.3
petunidin-3-(6″-*O*-acetyl)-glucoside	17.0 ± 0.1	32.6 ± 0.2
Σ petunidin	150.3 ± 1.1	296.1 ± 2.2
cyanidin-3-glucoside	62.9 ± 0.5	140.0 ± 1.1
cyanidin-3-galactoside	21.8 ± 0.6	48.5 ± 1.3
cyanidin-3-arabinoside	17.4 ± 0	41.5 ± 0.0
Σ cyanidin	102 ± 1.1	230.0 ± 2.4
Sum of ACNs	1295.5 ± 9.3	2621.5 ± 18.7

**Table 2:** Sequence of primers and probes used for real-time PCR.

Gene	GenBank Accession No.	Primer and TaqMan Probe Sequence
E-selectin	M30640	Forward primer: 5′-CCCGTGTTTGGCACTGTGT-3′
		Reverse primer: 5′-GCCATTGAGCGTCCATCCT-3′
		TaqMan probe: 5′-Fam-CAAGTTCGCCTGTCCTG-Tamra-3′
VCAM-1	M60335	Forward primer: 5′-GGGAAGCCGATCACAGTCAA-3′
		Reverse primer: 5′-ATGAGATGATCTCCTTTCAGTAAGTCTATC-3′
		TaqMan probe: 5′-Fam-TCAGTTGCTGATGTATACCCATTTGA- CAGGC-Tamra-3′
ICAM-1	NM_000201	Forward primer: 5′-GCAGACAGTGACCATCTACAGCTT-3′
		Reverse primer: 5′-CTTCTGAGACCTCTGGCTTCGT-3′
		TaqMan probe: 5′-Fam-CCGGCGCCCAACGTGATTCT-Tamra-3′
IL-6	M54894	Forward primer: 5′-GGTACATCCTCGACGGCATCT-3′
		Reverse primer: 5′-GTGCCTCTTTGCTGCTTTCAC-3′
		TaqMan probe: 5′-Fam-TGTTACTCTTGTTACATGTCTCCTTTCTCAGGGCT-Tamra-3′
IL-8	M28130	Forward primer: 5′-AGCTGGCCGTGGCTCTCT -3′
		Reverse primer: 5′-TTTAGCACTCCTTGGCAAAACTG -3′
		TaqMan probe: 5′-Fam-CAGCCTTCCTGATTTCTGCAGC-TCTGTG-Tamra-3′
NF-κB (RelA)	NM_62399	Forward primer: 5′-AGCACAGATACCACCAAGACCC-3′
		Reverse primer: 5′-CCAGGGAGATGCGCACTG-3′
		TaqMan probe: 5′-Fam-CATCAAGATCAATGGCTACACGGACCAGG-Tamra-3
β-actin	NM_002046	Forward primer: 5′-CCACATCGCTCAGACACCAT-3′
		Reverse primer: 5′-GTGACCAGGCGCCCAATA-3′
		TaqMan probe: 5′-Fam-AGGTCGGAGTCAACGGATTTGG-Tamra-3′

**Table 3:** Degradation rates of ACNs as a function of incubation time in Caco-2 cells ^1^.

MO	Phase	Time (min)	Del-3-glc	Cy-3-glc	Pet-3-glc	Peo-3-glc	Mal-3-glc
*H. alvei*	(1)	0	1.87 ± 1.00	0.48 ± 0.01	2.49 ± 0.96	8.26 ± 1.05	12.98 ± 1.25
		120	0.11 ± 0.00	0.45 ± 0.03	0.48 ± 0.45	2.50 ± 0.12 *	4.41 ± 1.05 *
		240	0.03 ± 0.00	0.39 ± 0.05	0.35 ± 0.10	2.32 ± 0.52 *	4.04 ± 0.95 **
	(2)	0	1.36 ± 0.59	067 ± 1.15	2.07 ± 0.61	6.50 ± 0.97	11.25 ± 2.19
		120	0.10 ± 0.27	0.63 ± 1.02	0.77 ± 0.35	2.96 ± 1.05	5.65 ± 1.05
		240	0.05 ± 0.45	0.45 ± 0.44	0.53 ± 0.25	2.34 ± 0.16 *	4.71 ± 0.15 **
	(3)	0	0.11 ± 0.09	0.45 ± 0.25	0.48 ± 0.13	2.24 ± 0.19	4.41 ± 2.56
		120	0.07 ± 0.06	0.41 ± 0.35	0.61 ± 0.36	0.14 ± 0.17	4.96 ± 2.16
		240	0.01 ± 0.05	0.36 ± 0.38	0.40 ± 0.46	0.20 ± 0.17	4.04 ± 1.94
*E. coli*	(1)	0	1.16 ± 0.52	0.44 ± 0.21	1.45 ± 0.32	8.69 ± 2.04	14.33 ± 3.86
		120	nd	0.39 ± 0.16	0.31 ± 0.09	4.21 ± 1.49	7.57 ± 1.09
		240	nd	0.34 ± 0.45	0.27 ± 0.06	3.84 ± 0.38 *	7.13 ± 0.68 *
	(2)	0	0.03 ± 0.00	0.03 ± 0.00	0.06 ± 0.00	0.37 ± 0.00	0.50 ± 0.00
		120	nd	nd	nd	0.32 ± 0.00	0.64 ± 0.00
		240	nd	nd	nd	0.28 ± 0.00	0.53 ± 0.00
	(3)	0	nd	nd	nd	0.11 ± 0.00	0.16 ± 0.00
		120	nd	nd	nd	0.10 ± 0.00	0.17 ± 0.00
		240	nd	nd	nd	0.10 ± 0.00	0.19 ± 0.00
*E. faecalis*	(1)	0	2.09 ± 1.05	2.09 ± 0.28	2.93 ± 1.06	10.58 ± 2.00	15.83 ± 3.55
		120	0.09 ± 0.54	0.76 ± 0.25	0.82 ± 0.15	4.03 ± 1.01	7.13 ± 2.49
		240	nd	0.63 ± 0.37	0.64 ± 0.35	3.77 ± 0.66 *	6.30 ± 1.49 **
	(2)	0	0.30 ± 0.21	1.12 ± 2.25	1.04 ± 0.54	6.18 ± 2.41	9.21 ± 2.97
		120	nd	0.45 ± 0.65	0.35 ± 0.55	2.88 ± 1.05	4.90 ± 2.49
		240	nd	0.31 ± 0.35	0.15 ± 0.49	2.14 ± 1.00	3.84 ± 1.19 *
	(3)	0	0.24 ± 0.00	0.91 ± 0.00	0.88 ± 0.00	5.22 ± 2.64	7.80 ± 5.05
		120	nd	0.35 ± 0.00	0.26 ± 0.00	2.45 ± 2.41	4.11 ± 2.05
		240	nd	0.23 ± 0.00	0.14 ± 0.00	1.67 ± 1.05	2.96 ± 1.05

^1^ MO-fermented ACN supernatants from different time points (lag-phase (1), log-phase (2) and stat-phase (3)) of *H. alvei*, *E. coli* and *E. faecalis* were exposed to the apical compartment of the co-culture system. Then, after a 0-, 120- and 240-min incubation time, concentrations of ACNs and metabolites were determined in apical and basolateral compartments as described in the Materials and Methods. Values are given as the means ± SD (*n* = 3, each done in duplicates). Differences between ACN fermentation and the initial ACN concentration in the growth media were significant at * *p* < 0.05 and ** *p* < 0.01. (Del-3-glc = delphidine-3-glucoside, Cy-3-glc = cyanidine-3-glucoside, Pet-3-glc = petonidine-3-glucoside, Peo-3-glc = peonidin-3-glucoside, Mal-3-glc = malvidine-3-glucoside>, nd = not detected).
